# Pilot survey reveals ophidiomycosis in dice snakes *Natrix tessellata* from Lake Garda, Italy

**DOI:** 10.1007/s11259-023-10129-7

**Published:** 2023-04-29

**Authors:** Daniele Marini, Matteo R. Di Nicola, Veronica Crocchianti, Tommaso Notomista, Daniel Iversen, Luca Coppari, Michela Di Criscio, Vanessa Brouard, Jean-Lou C.M. Dorne, Joëlle Rüegg, Maria Luisa Marenzoni

**Affiliations:** 1 Department of Organismal Biology, Evolutionary Biology Centre, Uppsala University, Norbyvägen 18A, Uppsala, 75236 Sweden; 2 Department of Veterinary Medicine, University of Perugia, Via San Costanzo 4, Perugia, 06126 Italy; 3 Unit of Dermatology, IRCCS San Raffaele Hospital, Via Olgettina 60, Milan, 20132 Italy; 4 Asociación Herpetológica Española, Apartado de correos 191, Leganés, Madrid, 28911 Spain; 5 Service d’Anatomie Pathologique, VetAgro Sup, Campus Vétérinaire, 1 Avenue Bourgelat, Marcy l’Etoile, 69280 France; 6 Via Varano 1, Castellammare di Stabia, 80053 Italy; 7 Viale Giovanni Prati, Riva del Garda, 38066 Italy; 8 Studio Naturalistico Hyla s.r.l, Via Baroncino, 11, Tuoro sul Trasimeno, PG 06069 Italy; 9 Methodology and Scientific Support Unit, European Food Safety Authority (EFSA), Via Carlo Magno 1A, Parma, 43126 Italy

**Keywords:** Europe, *Ophidiomyces ophidiicola*, Ophidiomycosis, SFD, Snake Fungal Disease, SYBR Green-based real-time PCR, Scale clipping

## Abstract

Ophidiomycosis is an emerging infectious disease caused by the fungus *Ophidiomyces ophidiicola* (Oo). To date, Oo presence or associated disease condition has been recorded in wild and/or captive snakes from North America, Europe, Asia and Australia, but the data is still scarce outside the Nearctic. Although Italy is a country with a high snake biodiversity in the European panorama, and animals with clinical signs compatible with Oo infection have been documented, to date no investigations have reported the disease in the wild. Therefore, a pilot survey for the Italian territory was performed in conjunction with setting up a complete diagnostic workflow including SYBR Green-based real-time PCR assay for the detection of Oo genomic and mitochondrial DNA combined with histopathology of scale clips. Oo presence was investigated in 17 wild snake specimens from four different species. Four snakes were sampled in a targeted location where the mycosis was suspected via citizen science communications (i.e. North of the Lake Garda), whereas other ophidians were collected following opportunistic sampling. Oo genomic and mitochondrial DNA were detected and sequenced from all four Lake Garda *Natrix tessellata*, including three juveniles with macroscopic signs such as discolouration and skin crusts. From histopathological examination of scale clips, the three young positive individuals exhibited ulceration, inflammation and intralesional hyphae consistent with Oo infection, and two of them also showed the presence of arthroconidial tufts and solitary cylindrical arthrospores, allowing “Ophidiomycosis and Oo shedder” categorisation. For the remaining snake samples, the real-time PCR tested negative for Oo. This pilot survey permitted to localise for the first time Oo infection in free-ranging ophidians from Italy. Ophidiomycosis from Lake Garda highlights the need to increase sampling efforts in this area as well as in other northern Italian lakes to assess the occurrence of the pathogen, possible risk factors of the infection, its impact on host population fitness and the disease ecology of Oo in European snakes.

**Supplementary Information:**

The online version contains supplementary material available at 10.1007/s11259-023-10129-7.

## Introduction

Emerging infectious diseases (EIDs) have been defined as infectious diseases which have recently appeared in a population, and can increase in incidence or with an expanding range (Fisher 2018). EIDs can constitute a hazard for animal health and biodiversity since they can pose a serious threat to organisms and can even cause local and global extinctions (Hoberg and Brooks 2015). With regards to snakes, ophidiomycosis, also known as snake fungal disease and caused by the keratinophilic fungus *Ophidiomyces ophidiicola* (Oo, formerly *O. ophiodiicola*), has raised concerns in recent years as an EID (see Di Nicola et al. 2022a). Even if the severity of its impact on snake populations is still unclear (McKenzie et al. 2021; Di Nicola et al. 2022a), Oo presence or associated mycosis has currently been recorded in wild and/or captive snakes from North America, Europe, Asia and Australia (e.g. Sigler et al. 2013; Lorch et al. 2016; Franklinos et al. 2017; Sun et al. 2021; Takami et al. 2021).

Field monitoring of ophidiomycosis is complex since snakes are usually elusive animals, and are difficult to observe. Furthermore, their activity is influenced by climatic-environmental conditions. Trap systems (e.g. pitfall and funnel traps) require a considerable field effort by the operators, and often prove to be ineffective, especially in areas with relatively low densities of individuals or in the case of unsuitable climatic conditions (see Turner 1978; Ward et al. 2017; Di Nicola et al. 2021). Hence, the low detectability and the difficulty of sampling snakes in the wild hampers surveillance of Oo at the population level, particularly in the short-term. Consequently, the actual effect of this disease on snake population trends may be challenging to assess.

A recent review has provided a world map of ophidiomycosis on a global scale, reviewing the cases of detection and infection of Oo published in the scientific literature up to June 2021 (Di Nicola et al. 2022a): Oo has been reported for 62 snake species and in 11 different countries (in 4 of which the fungus was only detected from snakes held in captivity). Since then, further investigations have been performed, and regarding newly reported species and/or countries, Oo presence was also confirmed in: captive specimens of *Acrochordus granulatus* imported from Indonesia to a Russian zoo (Ovchinnikov et al. 2021); free-ranging *Python bivittatus* from Hong Kong, China (Grioni et al. 2021); different free-ranging European snakes (i.e. *Coronella austriaca*, *Hierophis viridiflavus*, *Natrix maura*, *N. natrix*, *Zamenis longissimus* and *Vipera nikolskii*) in several further states (i.e. Austria, France, Germany, Hungary, Poland and Ukraine) (Blanvillain et al. 2022). Overall, the presence of Oo has been detected mainly in North America, while in Europe, Asia and Oceania data is still scant, and reliable information is lacking for Central/South America and Africa. The actual knowledge on Oo detection and/or infection in European free-ranging snakes is still limited. Oo detections have only been reported in 9 European countries (i.e. Austria, Czech Republic, France, Germany, Hungary, Poland, Switzerland, United Kingdom and Ukraine) and in 9 out of 57 European snake species (according to Di Nicola et al. 2022b; i.e. *Coronella austriaca*, *Hierophis viridiflavus*, *Natrix helvetica, N. maura*, *N. natrix*, *N. tessellata*, *Zamenis longissimus*, *Vipera berus* and *V. nikolskii*) (see Franklinos et al. 2017; Meier et al. 2018; Blanvillain et al. 2022; Schüler et al. 2022). Furthermore, Origgi et al. (2022) found ophidiomycosis in preserved museum specimens: *Natrix helvetica* (dated 2001), *N. natrix* (dated 1963) and *N. tessellata* (dated 1961) from Switzerland and two *N. helvetica* (dated 1959 and 1967) from Italy. The samples generically labelled as Italian do not have a more precise geographical indication.

Italy constitutes one of the most herpetological diverse countries in Europe (Nania et al. 2022), hosting about 59 species of reptiles (Sindaco and Razzetti 2021): it includes 22 species of snakes that represent 39% of the 57 European ophidian species (including allochthonous taxa) so far reported (Di Nicola et al. 2022b). Nonetheless, reports of screenings in Italy for Oo have been scarce to date, only being conducted in two cases; in both instances, Oo was not detected (i.e. Cocullo and Pretoro “Serpari” festivals, local events involving wild snakes – Marini et al. 2022a, b). Moreover, a few observations of snakes with clinical signs compatible with ophidiomycosis appeared in a single publication (Meier et al. 2018) or in reports from citizen scientists in social media, and none of these observations were confirmed with testing. Hence, no prospective studies recording Oo presence/infection in Italian free-ranging snakes have been reported so far.

The aim of the present study was to perform a pilot survey to investigate Oo presence and infection in snake species from Italy while obtaining a proof-of-concept intended to optimise a complete experimental diagnostic workflow with rapid execution time and, in particular, at low cost. An additional objective was to collect a range of different samples and apply feasible sampling methods to allow Oo investigation in the most efficient and versatile manner.

## Materials and methods

### Study area and sampling

Since 2015, some *Natrix tessellata* and *N. helvetica* individuals with dysecdysis, crusty lesions and swellings have come to the attention of the authors through citizen science photographs and consecutive personal surveys. These snakes have been spotted on the shores of northern Italian lakes such as Maggiore, Como, Annone and, in particular, Garda. Considering the relevance of the observations coming from the northern portion of Lake Garda (e.g. *N. tessellata* in Fig. S1), Riva del Garda municipality was selected as the priority area for the pilot study. At the same time, samples from live snakes and road-killed specimens from other species and locations across Italy were tested to increase the sample size and to compare Oo screening results.

**Fig. 1:**
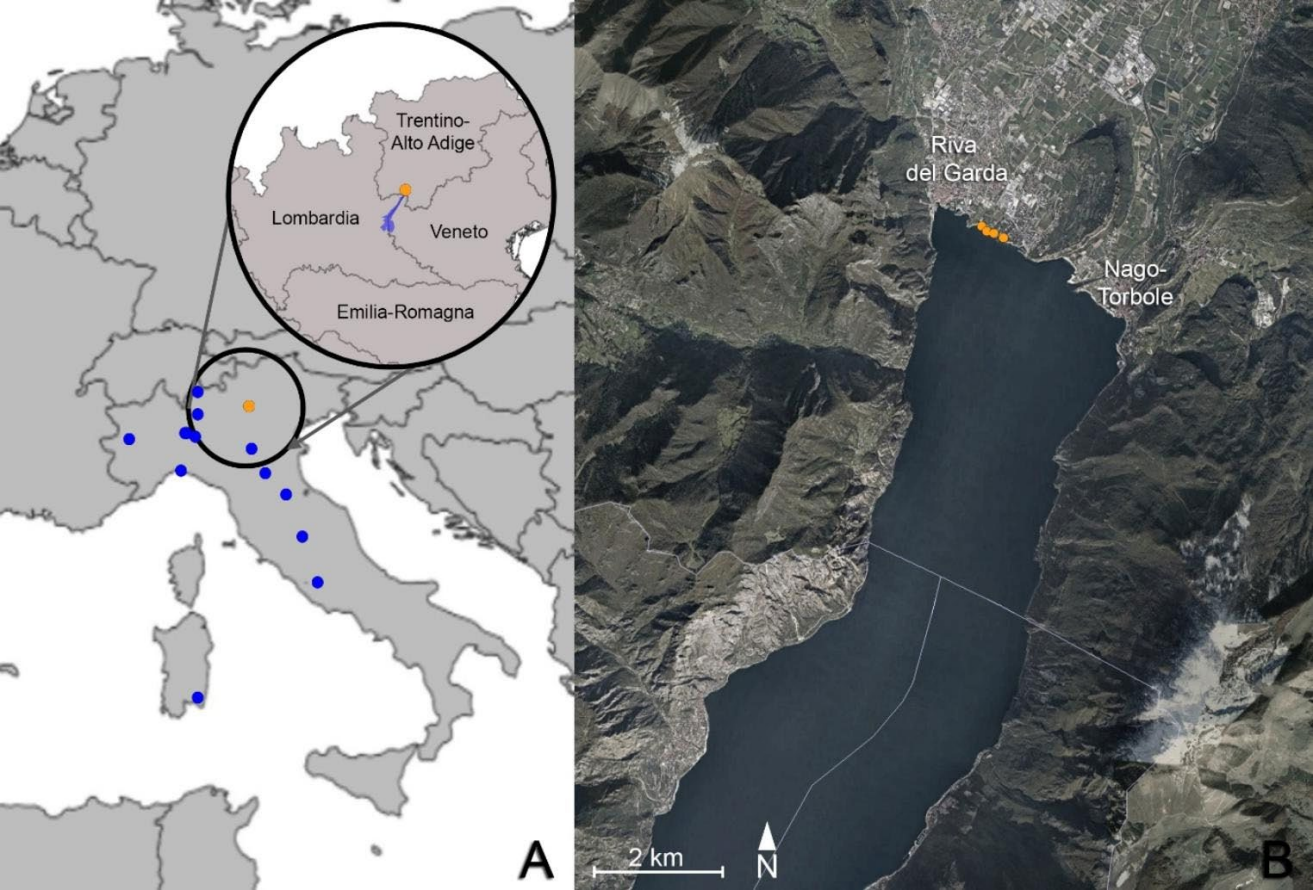
Study area. (**A**) Samples in the European context: orange dot represents Lake Garda samples; blue dots represent additional samples from other Italian localities. Inset: Detail of Garda’s samples within Italian regions’ borders. (**B**) Focus on the interested Lake Garda coast with delimited regional borders. Orange dots represent the 4 *Natrix tessellata* from Riva del Garda (Province of Trento, Italy). Map source: Google Earth (modified)

The Lake Garda field survey was conducted along a 1165 m stretch of coast (municipality of Riva del Garda, Autonomous Province of Trento; coordinates: 45°52’51"N, 10°50’58” E; elevation: 62 m a.s.l. - Fig. 1). Specifically, the visual encounter survey took place along the rock armour, occupying 56% (i.e. 688 m) of the portion of the surveyed stretch, otherwise occupied by a sandy beach. This artificial rocky shore was selected as particularly suitable habitat for the presence of *N. tessellata* (Scali et al. 2011). The survey was performed on the 2^nd^ March 2021, a late winter day with a relatively mild climate (i.e. clear weather; T min = 0°C, T max = 16°C; average wind speed = 6 km/h). Each captured snake was carefully examined, measured and photographed with a focus on investigating any possible lesions. Dry swabbing was then performed with a single sterile cotton-tipped applicator for each individual for ten repetitions on the dorsal scales, ventral scales and head region in order to cover all the skin surface. Finally, the same applicator was also rubbed ten times on each identified lesion. The entire procedure was repeated three times, for a total of three dry swabs per snake. Dry swab tips were placed in 1.5 ml tubes, subsequently stored at -20°C. Superficial scale clipping was performed on the skin close to the main lesions (if any), while avoiding reaching the subcutaneous tissue, using disposable sterile surgical scissors (Iris scissors curved 11.5 cm, Peha®-instrument). All tissue samples were then fixed in 96% ethanol. Following these procedures, each snake was released at the precise location where it was found to minimise the effect of sampling on the snakes’ behaviour.

Additional individuals from other Italian localities were opportunistically sampled to increase the sample size for the analysis (Fig. 1). Tissues from road-killed specimens preserved in 96% ethanol or frozen, and dry swabs from live snakes were collected. In latter cases, swabbing was performed as described above, whereas for dead specimens, when possible (i.e. clinically affected individuals), tissues showing gross lesion(s) were preferred over apparently healthy skin.

### Molecular analysis and sequencing

Molecular analysis was applied for screening Oo presence and confirming the fungal identification using 3 swabs and/or tissue samples – scale clips or tissues from road-killed specimens – collected from each snake (see Table S1).

DNA was extracted from dry swabs and tissues according to the method by Hyatt et al. (2007) and Franklinos et al. (2017). In brief, 100 µl (for dry swabs) or 50 µl (for tissues) of PrepMan Ultra Sample Preparation Reagent (ThermoFisher, Carlsbad, CA, USA) and 50 mg of 0.5 mm diameter zirconium oxide beads were added to 1.5 ml tubes containing the samples. Homogenization for 60 s with Bullet Blender Storm 24 (Next Advance, Inc. New York, USA) followed by a centrifugation (30 s at 13 000 RPM) was repeated twice. Then, samples were heated 12 min at 95°C with a thermoblock, cooled for 5 min and centrifuged again (30 s at 13 000 RPM). Twenty to 30 µl of supernatant was recovered and DNA concentration (Table S1) and quality was evaluated with Nanodrop 2000c spectrophotometer (ThermoFisher, Carlsbad, CA, USA). When applicable, an aliquot of the extracts was diluted to a concentration of 25 ng/µl. Successively, original extracts and eventual aliquots were stored at -20°C or directly used. If all swab samples from an individual resulted negative at the real-time PCR, DNA was also extracted from an available scale clip stored in 96% ethanol.

The DNA from each swab or tissues was first run in triplicate with a real-time PCR for the internal transcribed spacer 2 (ITS2) within the ribosomal RNA (rRNA) gene complex of Oo. When the samples where positive for ITS2, the DNA from a single positive swab was analysed in triplicate to confirm the presence of Oo with (i) a real-time PCR targeting mitochondrial Oo NADH dehydrogenase subunit 1 (nad1) and (ii) a broad-range panfungal conventional PCR targeting genomic D1-D2 region of the large subunit of the rRNA gene.

Real-time PCRs specific for genomic and mitochondrial Oo DNA were performed using primer sets targeting (i) ITS2 and (ii) nad1, designed by (i) Bohuski et al. (2015) and (ii) Lorch et al. (2021), respectively (Table S2). A real-time PCR using SYBR Green was carried out for both primer sets. Each 10 µl reaction used the following volumes of reagents: 5 µl of iQ SYBR Green Supermix (Bio-Rad Laboratories Inc. Hercules, CA, USA); 16–100 ng of DNA in a volume of 4 µl (original concentrations in Table S1); 0.7 µl of nuclease-free water, 0.3 µl of 10 µM suspension of forward and reverse primers. A CFX384 Touch Real-Time PCR Detection System (Bio-Rad Laboratories Inc. Hercules, CA, USA) was used to amplify products of both primer sets with the same following cycling conditions (used also by Bohuski et al. 2015 in the original TaqMan assay – see Fig. S2): 3 min at 95°C, then 40 cycles of 3 s at 95°C, and 30 s at 60°C.

Real-time PCR results and melting curves were analysed using Bio-Rad CFX Maestro software to ensure enrichment of only one product. The efficiency of the real-time PCR assay for the ITS2 Oo gene was tested using a positive control of DNA extracted from tissue (Fig. S3) kindly provided by F.C. Origgi. Negative controls were established using nuclease-free water instead of DNA template. The absence or presence of Oo DNA for diagnostic purposes was discriminated by analysing the final relative fluorescence units (RFUs) for the sample wells via Bio-Rad CFX Maestro software. Samples considered positive had an RFU value greater than the average RFU value of the negative controls plus the cut off value. The cut off value was calculated by adding the tolerance (setting the Percentage of Range at 20) to the average of the negative controls. The number of cycles used to calculate the average end-point RFU in the setting was 5 (End Cycle to Average). Moreover, threshold cycle (Ct) values across real-time PCR runs were evaluated to confirm the presence of Oo DNA.

Primers from Franklinos et al. (2017 - Table S2) were used to carry out the broad-range panfungal conventional PCR targeting genomic D1-D2 region. The latter genomic fragment was amplified using the PyroMark PCR kit (Qiagen − 12.5 µl of PyroMark PCR Master mix, 1 µl of 25 mM MgCl_2_, 7 µl nuclease-free water and 2.5 µl of CoralLoad Concentrate) together with 0.5 µl (500nM) forward primer, 0.5 µl (500nM) reverse primer, and 1 µl of DNA template, for a total 25 µl per reaction incubated at the following conditions: 95°C for 15 min; 40 cycles of 94°C for 30 s, 58°C for 30 s, 72°C for 30 s; final extension of 72°C for 10 min. Finally, the products were run on agarose gel for visualisation and size determination.

A subset of amplicons from real-time and conventional PCR were separated on agarose gels to be checked for the expected size. Then, specific bands were excised from the agarose gel and extracted using QIAquick® Gel Extraction Kit according to the manufacturer’s instructions. Otherwise, after the inspection of DNA fragments, amplification products from the well were directly purified with QIAquick® PCR Purification Kit according to the manufacturer’s instructions. Purified PCR products were directly Sanger sequenced by a commercial service (Macrogen, The Netherlands) on both strands using the same primers employed for previous amplifications. The resulting sequences were edited and assembled using BioEdit software and compared with the ones deposited in GenBank using the Basic Local Alignment Search Tool (BLAST) software (https://blast.ncbi.nlm.nih.gov/Blast.cgi). Moreover, some selected obtained sequences were submitted to the GenBank database.

### Histopathology

Histological examination was carried out in cases for which Oo was detected by molecular means to confirm infection, when tissue was still available. Tissues stored in 96% ethanol were further fixed in 10% neutral buffered formalin for 24 h, and then routinely processed into paraffin blocks with a longitudinal orientation. Sections measuring 5 μm were cut, stained with Hematoxylin-Eosin (HE), Periodic acid–Schiff (PAS) and Grocott’s methenamine silver, and examined under light microscopy. Additionally, selected slides were scanned with Leica Aperio AT2 (magnification: 20X, 0.75 Numerical Aperture, 2X optical magnifier) and used for morphometric measurements or snapshot capture with QuPath software (version 0.3.2).

## Results

### Sampling data and gross findings

**Fig. 2:**
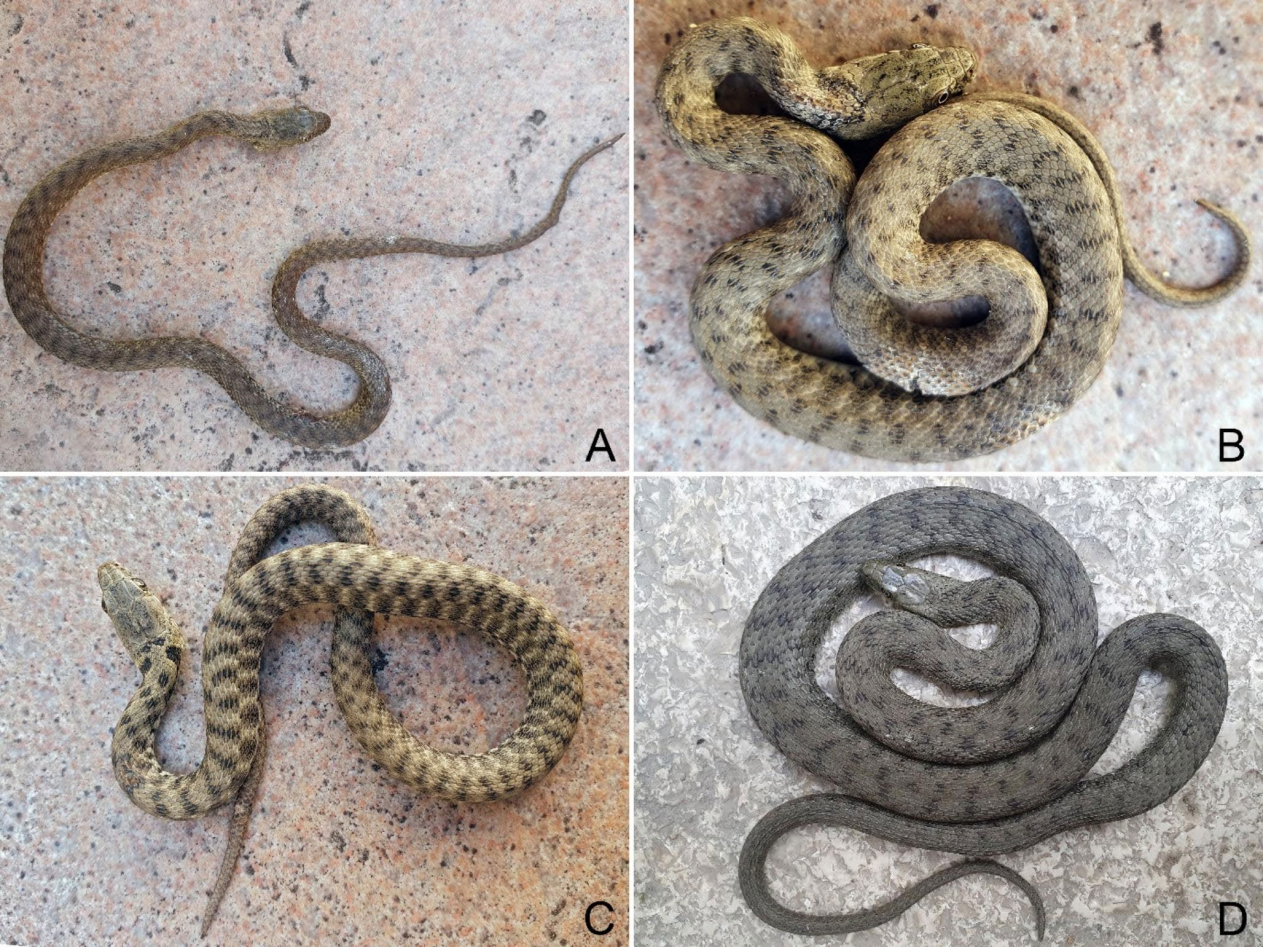
*Natrix tessellata* individuals observed on the 2^nd^ of March 2022 and sampled for Oo infection. (**A**) NT1, juvenile. (**B**) NT2, juvenile. (**C**) NT3, juvenile. (**D**) NT4, adult. Photocredits: Matteo R. Di Nicola

**Fig. 3:**
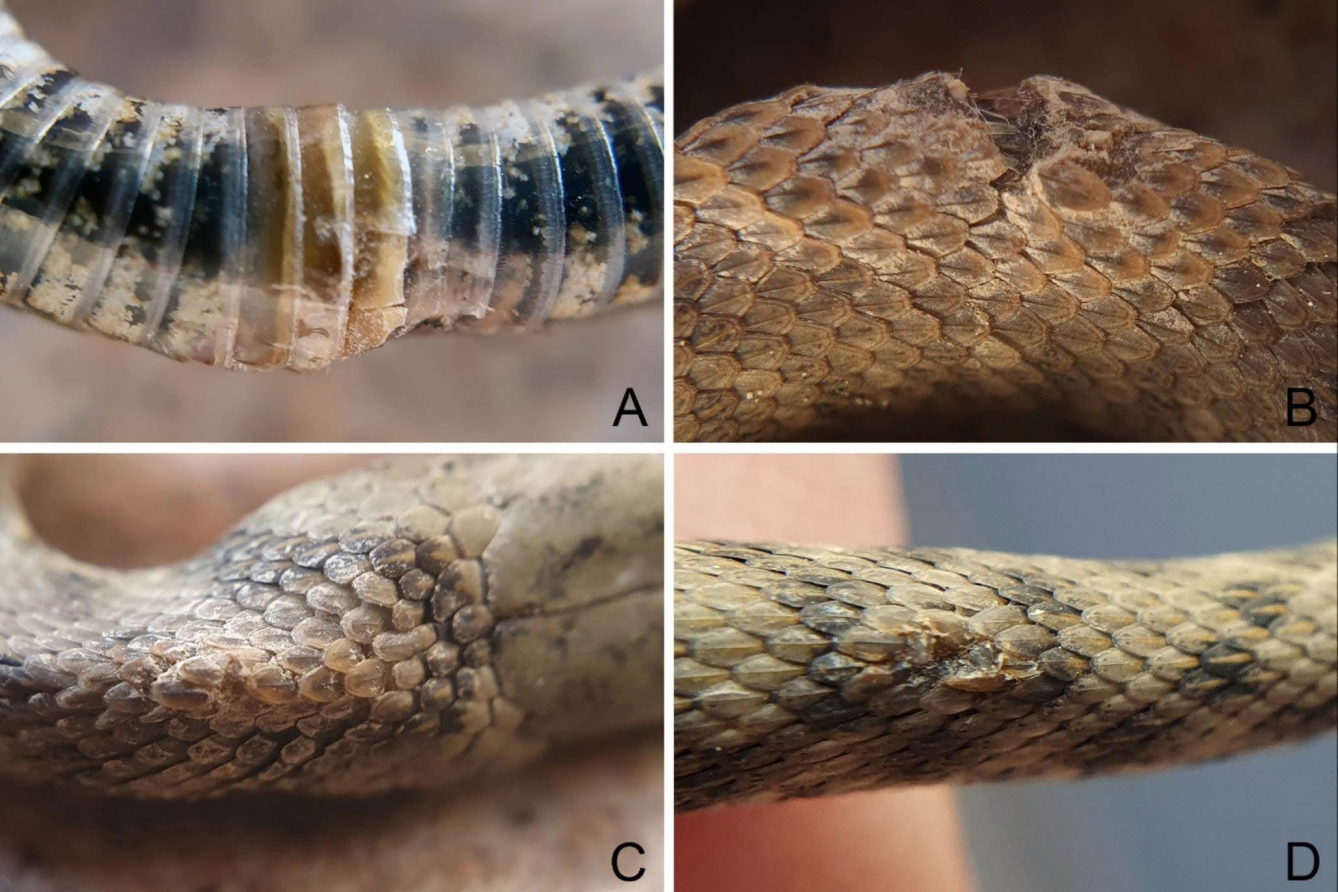
Main lesions consistent with Oo infection observed in the three symptomatic juvenile *Natrix tessellata*. (**A**) NT1, Focal yellowish, discoloured and erosed ventral scales. (**B**) NT2, Focal skin ulceration with a dark necrotic aspect in the mid region of the body. (**C**) NT2, Dorsal scales close to the head region: focal crust displacing a scale. (**D**) NT3, Crusts taking the place of missing scales in the dorsal side of the tail. Photocredits: Matteo R. Di Nicola

On 2^nd^ March 2021, from 11 am onwards, a field survey was conducted in the above-mentioned stretch of Lake Garda coast and four *Natrix tessellata* individuals, namely NT1, NT2, NT3 and NT4, were encountered and found active on such coastal area (Fig. 1B). The first three snakes had a total length of 22.1 cm, 21.2 and 21.6 cm, and likely represented young individuals born in the previous summer period (Scali et al. 2011; Di Nicola et al. 2021 - Fig. 2A, B, C); the fourth individual was a 54 cm adult (Fig. 2D). Clinical signs consistent with Oo infection (i.e. discolouration, displaced scales, skin ulcerations and crusts - Fig. 3) were found in the three juveniles (NT1, NT2, NT3), whereas the adult individual (NT4) did not show any clinical sign.

Additional samples originating from 13 wild snakes found in other Italian localities were included in the analysis (Table 1, S3; Fig. 1A). Amongst these ophidians, eight were road-killed specimens (four preserved in 96% ethanol and four frozen), while the other five were alive and sampled using dry swabs. These additional snakes came from seven Italian regions and belonged to 4 different taxa with an age range spanning from juvenile to adult. Four out of these 13 ophidians were showing gross signs consistent with Oo mycosis (Table 1, S3).

**Table 1:** Snake samples included in the study. See detailed information in Table S3. + means positive. - means negative. N.P. means not performed

ID code	Species	Date	Locality	Age class	Finding condition	Gross signs	Molecular results	Histology
CG21	*Coronella girondica*	‘21-10-05	Guzzano (Emilia-Romagna)	Juvenile	Dead	Y	-	N.P.
CG01	*Coronella girondica*	‘21-11-11	Genova (Liguria)	Sub-adult	Dead	N	-	N.P.
CG02	*Coronella girondica*	‘21-02-02	Bagno di Romagna (Emilia-Romagna)	Adult	Live	N	-	N.P.
HV03	*Hierophis viridiflavus*	‘21-05-10	Giussago (Lombardy)	Sub-adult	Dead	N	-	N.P.
HVGU	*Hierophis viridiflavus*	‘21-09-29	Guidonia Montecelio (Latium)	Adult	Dead	Y	-	N.P.
HVPG	*Hierophis viridiflavus*	‘21-05-13	Marsciano (Umbria)	Juvenile	Live	Y	-	N.P.
BSS	*Hierophis viridiflavus*	‘21-05-19	Sorico (Lombardy)	Adult	Live	Y	-	N.P.
HV02	*Hierophis viridiflavus*	‘21-02-09	Moglia (Lombardy)	Adult	Dead	N	-	N.P.
HV01	*Hierophis viridiflavus*	‘21-03-10	Villanterio (Lombardy)	Adult	Dead	N	-	N.P.
NHCE	*Natrix helvetica cetti*	‘21-04-03	Sinnai (Sardinia)	Adult	Live	N	-	N.P.
NH02	*Natrix helvetica sicula*	‘21-09-01	Montevecchia (Lombardy)	Sub-adult	Dead	N	-	N.P.
NH01	*Natrix helvetica sicula*	‘21-04-25	Caselette (Piedmont)	Sub-adult	Live	N	-	N.P.
NH03	*Natrix helvetica sicula*	‘21-07-01	Giussago (Lombardy)	Adult	Dead	N	-	N.P.
NT1	*Natrix tessellata*	‘21-03-02	Riva del Garda (Province of Trento)	Juvenile	Live	Y	+	+
NT2	*Natrix tessellata*	‘21-03-02	Riva del Garda (Province of Trento)	Juvenile	Live	Y	+	+
NT3	*Natrix tessellata*	‘21-03-02	Riva del Garda (Province of Trento)	Juvenile	Live	Y	+	+
NT4	*Natrix tessellata*	‘21-03-02	Riva del Garda (Province of Trento)	Adult	Live	N	+	N.P.

### Molecular results

Oo genomic (ITS2) and mitochondrial (nad1) DNA was detected in all dice snakes from Garda lake (NT1, NT2, NT3, NT4). PCR results are shown in Table 1, S3 and S4. In all the young dice snakes (NT1, NT2, NT3), 2 out of 3 swabs (66.6%) were positive in triplicate or duplicate for the ITS2 gene, whereas for the adult (NT4) Oo ITS2 was detected only from the scale clip and all the 3 swabs tested negative. Mitochondrial nad1 and genomic D1-D2 region were successfully amplified from one random DNA template that was PCR-positive for the ITS2 target; amplicons were of the expected size in all instances. The RFU averages at the end of the PCR reactions from ITS2 for positive samples ranged from 1070 to 1960, while the ones from the positive control ranged from 1635 to 2365 (Table S4). For the nad1 gene, these values ranged from 496 to 1080 for the samples with a range of 1118–1973 for the positive control (Table S4). The averages of the Ct values in positive samples for the ITS2 were equal to or below 29.67 whereas positive controls reached the maximum average Ct value of 24.20. In contrast, the maximum average Ct values of positive samples for nad1 was 31.66 while the positive control average Ct values were 26.65 or 29.63 (Table S4). Averages of melting temperatures were equal to or lower than 0.5 or 1°C compared to the ones of positive controls (ITS2: 84°C; nad1: 73.5°C) (Table S4). The melting curves and peaks showed the amplification of only one product and the amplicons’ size obtained on agarose gel was consistent with the expected product length.

Pairwise genetic distance of the obtained sequences with other deposited Oo sequences in GenBank (first Oo sequence in GenBank) varied between 96.43% and 100% (Table S5), except for D1-D2 locus from NT1 for which we obtained a sequence consistent with *Cladosporium* sp. (98.99% of homology with *C. sphaerospermum* and *C. halotolerans*; Table S5). Representative sequences (one sequence for each target region that showed 100% homology with Oo) were deposited in the NCBI Nucleotide database under accession numbers OQ612704, OQ613488 and OQ607750 (see Table S5). The sequence of the probe marked with 6-carboxyfluorescein (FAM) and Black Hole Quencher®-1 (BHQ-1) used to detect the Oo ITS2 gene in the original TaqMan real-time PCR system by Bohuski et al. (2015) was always present in our ITS2 sequences (Table S5).

ITS2 real-time PCR for the additional samples from outside Lake Garda (Table 1, S3) were all negative, hence no further analyses were performed.

### Histopathological evaluation

**Fig. 4:**
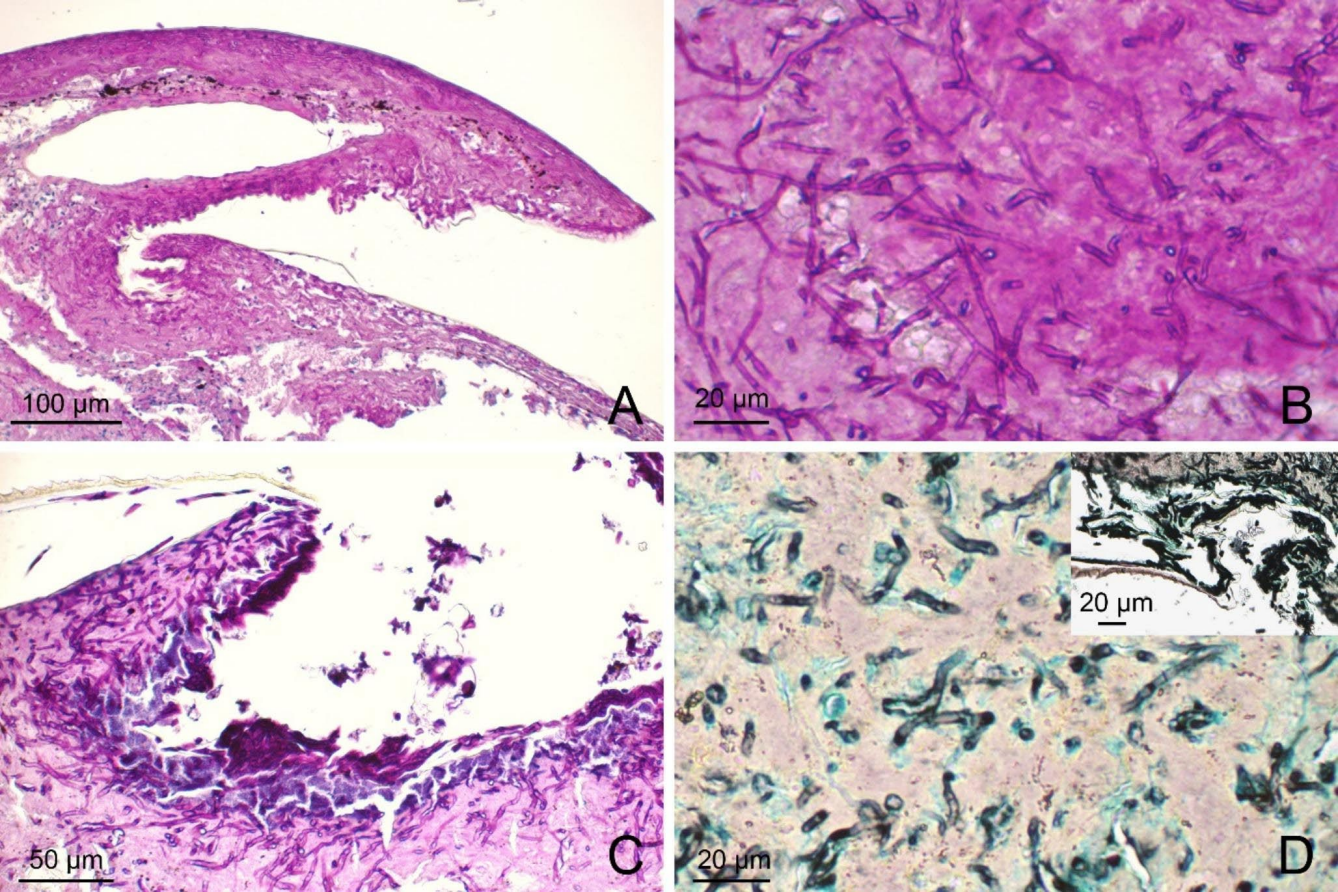
Histopathological features of ophidiomycosis in juvenile dice snakes. (**A**) NT1, Longitudinal section of clipped scale showing necrosis, fibrin, heterophils and a myriad Oo hyphae dispersed in the necrotic tissue, PAS. (**B**) NT1, Hyaline pauciseptate hyphae showing parallel walls, non-dichotomous acute-angle branching and transverse septa. PAS. (**C**) NT2, Longitudinal section of clipped scale with erosed epidermis, basophilic bacterial colonies, arthroconidial tufts and underlying numerous hyphae, PAS. **D**, NT1. Hyphae consistent with Oo, Grocott. Inset: NT2. Arthroconidiation on the air-tissue interface, Grocott

Scale clips from NT1, NT2 and NT3, deemed as positive for Oo based on the molecular screening results, were used for histopathological evaluation. The NT4 scale clip was used for molecular analysis and further tissue was not available for histopathology. In the microscopic evaluation, gross lesions were correlated with multifocal inflammatory lesions extending from superficial to basal epidermal layers. The β, meso and α layers were surmounted and often effaced by a thick, eosinophilic, acellular, necrotic tissue intermingled with eosinophilic fibrin, karyorrhectic degenerated heterophils and cell debris (Fig. 4A, C, S3). A myriad of PAS/silver stain-positive fungal hyphae (2 to 5 μm in diameter) were found dispersed in the necrotic tissue. These hyphae were parallel walled, hyaline, pauciseptated and had non-dichotomous branching (Fig. 4B, D). The keratinocytes of the basal layer were hyperplastic, often showing anisocytosis and disorganisation, being multifocally dysplastic and infiltrated by minimal to mild macrophages, pigmented macrophages and heterophils (Fig. S4). The air-lesion interface of NT1 and NT2 was populated by arthroconidial tufts and solitary cylindrical arthrospores (~ 2 × 4 μm – Fig. 4A, C, D inset).

## Discussion and conclusions

This study constitutes a pilot survey to detect the presence of Oo in Italian snakes, using photographic and citizen science evidence that highlighted clinical signs consistent with ophidiomycosis, particularly in snakes from northern Italian lakes (i.e. Lake Garda).

The use of real-time PCR with specific Oo primers in a SYBR Green-based assay yielded positive detections by means of RFUs (End-point) and Ct values analysis, which we were able to confirm by Sanger sequencing. In particular, the ITS2 products always contained the identical probe sequence designed for the original TaqMan real-time PCR by Bohuski et al. 2015 (Table S5). Moreover, we were able to successfully amplify both genomic and mitochondrial Oo DNA with the same volume of reagents and cycling conditions, and easily discriminate the products by their melting temperatures and curves/peaks (Table S4; Fig. S2). Thus, after further optimisation, this approach may be developed into a multiplex PCR able to simultaneously detect both ITS2 and nad1 in a single well. SYBR Green is more than 60% cheaper than TaqMan, because the reaction volumes for the real-time PCR assay can be reduced and for the intrinsic cost-effectiveness of the reagents (see Canessa et al. 2017). Furthermore, SYBR Green real-time PCR assay has already been validated and applied to the diagnosis of other emerging fungal pathogens in herpetofauna (Canessa et al. 2017; Costa et al. 2021). However, some authors consider that SYBR Green real-time PCR assay should not be used for clinical diagnosis of Onygenalean dermatomycoses because it does not allow for an acceptable product identification (Paré et al. 2020). SYBR Green is often considered to be less specific than TaqMan because it lacks a requirement for a specific probe to bind. In our case, it may not be an issue because the primers seem specific enough and, likely, the melting curves different from other non-target amplicons. Until further screen and direct comparison of sensitivity and specificity between SYBR Green and TaqMan assay is done, we suggest overcoming the latter issues with SYBR Green-based molecular detection of Oo by conducting careful inspection of melting curves, peaks and temperatures, as well as frequent sequencing of PCR products from positive samples.

Our study provides a proof-of-concept that Oo SYBR Green-based real-time PCR assay can generate reliable results, indicating its potential utility as an alternative method to the original TaqMan assay for Oo detection. However, further accurate validation is necessary to establish its performance characteristics (e.g. detection limit and reproducibility) and determine its level of agreement with the TaqMan assay. In addition, flank such tests with an implementation via fungal DNA absolute quantification (conversion of DNA concentration into number of copies or Genomic Equivalence) would allow broader, more frequent and affordable Oo screenings.

Amplicons resulting from PCR amplification of the three different Oo loci were sequenced. The sequences from all PCR positive individuals revealed that one or two regions among ITS2, nad1 and D1-D2 were 100% identical to existing Oo sequences in GenBank using BLAST (Table S5). On the other hand, some ITS2 or nad1 short amplicon sequences showed one or two gaps. Indeed, the quality outcome of Sanger sequencing targeting amplicons < 100 bp cannot be guaranteed (Crossley et al. 2020). Although it is beyond the aim of the current study, an “outer primer set” – amplifying a larger amplicon including the specific segment of interest – and/or cloning the amplicon within a vector would give longer and qualitatively better sequences and allow to further monitor the used real-time PCR assay (see Crossley et al. 2020).

Conventional broad-range PCR targeting genomic D1-D2 region can also be used to further confirm positive cases (e.g. Franklinos et al. 2017) and, as revealed in this screening, to have longer amplicons to sequence. Nonetheless, broad-range PCRs can result in amplification of common targets from different organisms, having DNA bands of the same length at electrophoresis that yield to detection of undesired or mixed sequences (overlapping peaks in the chromatograms – see Crossley et al. 2020). Therefore, this method might generate products not belonging to Oo, as observed in our study. As matter of fact, the amplified product from NT1 D1-D2 locus resulted in a *Cladosporium* sp. sequence, likely *C. spherospermum* or *C. halotolerans* (Table S5). We detected both Oo and *Cladosporium* from NT1, as also reported in a *Crotalus horridus* with concurrent ophidiomycosis by McBride and colleagues (2015). *Cladosporium* is a genus of saprophyte ascomycetes, occasionally able to induce infection in mammals (e.g. Huyan et al. 2012) or suspected to infect amphibians and reptiles (see McBride et al. 2015; Grassi et al. 2023). Nevertheless, pigmented hyphae consistent with *Cladosporium* morphology were not found at NT1 histology and this fungus might be a skin contaminant not related to the pathological signs.

In all positive cases resulting from swabbing of individuals with gross signs, 1 out of 3 replicate swabs did not show any ITS2 amplification, i.e. a 33.3% rate of false negative results. In the US, false negatives for Oo TaqMan real-time PCR after a single swab collection were very high, both from snakes with (73%) and without (97%) clinical signs (Hileman et al. 2018). Accordingly, Oo DNA was only amplified from NT4 tissue, an individual without macroscopic signs consistent with Oo infection and whose three swabs samples were negative for Oo. These high “failure” rates are likely mostly due to differences in swabbing techniques and superficiality of infection (Hileman et al. 2018). Unfortunately, we did not record the order of the cotton tip applicators used to sample the snakes, hence we cannot discuss further whether the negative results are due to the Oo genetic material being under the limit of detection, PCR inhibitors or to the swabbing method. Therefore, for future screening the authors recommend following and recording the order in which replicate dry swabs are collected. Moreover, especially when conducting initial Oo screening in a new location, it is advisable to collect multiple swabs as this may be beneficial to avoid false negatives in populations with unknown or low prevalence (Harding et al. 2022).

We performed histologic examination of tissues only when Oo was detected by molecular means, as this analysis was used as confirmation of disease and not as first line diagnosis or assessment for fungal presence. Scale clipping permitted us to investigate tissues of live snakes with histological methods, particularly the pathogen, its morphology (hyphae and conidia) and related inflammatory response. Histology of scales, belonging to a lesion or close to it, evidenced massive mycosis in that interested focal area. The morphology of fungal elements was compatible with *Ophidiomyces ophidiicola*. While PAS and Grocott’s methenamine silver stain highlighted fungal walls in the sections, we observed diaphanous/translucent cylindrical structures in the same location on HE slides. These structures were indicative of hyphal proliferation, but we were unable to confirm the presence of fungi as their walls were unstained. Despite the superficial sampling showing minimal or absent dermal and subcutaneous tissue in the sections, the fungal morphology was clear: a massive presence of characteristic hyphal and conidial structures on the superficial layers of epidermis. To our knowledge, scale clips have already been used for molecular detection of Oo (McKenzie et al. 2019; Harding et al. 2022), but not for histopathology. Scale clipping as a sampling method seems very versatile – the tissue can be used for molecular detection, histology and fungal culture – and minimally invasive when compared to other methods used in the field such as punch biopsies. However, it compromises the integrity of the integument and probably should be avoided in ophidians that are close to an imminent brumation (Di Nicola et al. 2022a). Having to choose a single fixative substance for each scale clip (due to the limited amount of biological material available), we opted for 96% ethanol, being a good compromise for both DNA conservation for molecular analysis and fixation for histology. Even if we encountered unavoidable artefacts during microscopy (e.g. vacuolation, quality loss of external layers - see Fig. 4), this fixation allowed for identification of the pathogen using histology and permitted us to have an additional back-up sample to perform a molecular-based assay. As a matter of fact, ethanol fixation of museum archival specimens allowed for successful Oo detection, both with molecular biology and histological methods (Lorch et al. 2021; Origgi et al. 2022).

Association between skin lesions and Oo molecular/histological presence suggests that clinical signs can be highly suggestive of cutaneous ophidiomycosis in dice snakes, as already observed in Eastern US snakes during their active period (Allender et al. 2016; McKenzie et al. 2019; Fuchs et al. 2020; Lind et al. 2022). A late winter day was chosen for the field survey in Lake Garda since it is a peri-brumation period for dice snakes in the considered area, and infected snakes are more likely to be actively thermoregulating and more easily encountered, while uninfected snakes may still be overwintering in hibernacula (Tetzlaff et al. 2017; Dillon et al. 2022; Lind et al. 2022).

To evaluate the susceptibility of various ophidian hosts, it is crucial to categorise cases of Oo detection and infection into specific classes. Following the three proposed diagnostic/classification criteria (i.e. Baker et al. 2019; Davy et al. 2021; Di Nicola et al. 2022a), the cases in our study may be graded in different manner. The classification of each case according to the three available criteria is provided in Table S3. Based on the classification proposed by Di Nicola et al. (2022a), NT1 and NT2 *N. tessellata* from Lake Garda are categorised as “Ophidiomycosis and Oo shedder”, NT3 as “Ophidiomycosis”, and NT4 as “Oo present”. This is based on the fact that, at microscopic level, propagules (i.e. arthroconidia) were detected in NT1 and NT2 sections (Fig. 4), meaning that these snakes were potentially able to spread and transmit the pathogen. In NT3, only hyphae and inflammation were found, which is a typical sign of Oo infection but not of the ability to spread the pathogen. NT4 scale clip yielded the molecular detection of Oo, but no histological analysis was performed to confirm the infection. Detection of Oo DNA in macroscopically intact tissue can be explained with fungal presence in deeper cutaneous layers due to a pre-clinical infection stage or residual pathogen subsequent to a healing process (Di Nicola et al. 2022a). Nevertheless, environmental contamination cannot be excluded even if skin swabs (limiting the sampling to the outermost portion of the tegument) from the same individual were negative. Samples from 13 ophidians belonging to other Italian areas tested negative at molecular detection and thus are excluded from the classification proposed by Di Nicola et al. (2022a). Based on the criteria by Baker et al. (2019), NT1 and NT2 are categorised as “Confirmed Ophidiomycosis”, NT3 as “Apparent Ophidiomycosis”, NT4 as “*Ophidiomyces* present”, and the four snakes with gross signs and negative Oo molecular detection as “Possible Ophidiomycosis”, whereas the nine snakes without macroscopic signs nor Oo molecular positivity are excluded from the classification. According to this grading system, NT3 is considered to be apparently affected by the disease because no arthroconidia were found in histological sections, unlike NT1 and NT2 where the propagule was identified confirming the ophidiomycosis. In case of Oo molecular negativity, the presence of gross signs discriminates from the possibility to be affected by the disease and the exclusion from the categorization by Baker and colleagues (2019). Based on the criteria by Davy et al. (2021), NT1, NT2 and NT3 are categorised as “Ophidiomycosis”, NT4 as “Detected”, and the remaining 13 ophidians negative at Oo molecular detection (with or without gross signs) as “Not detected”. The histopathological finding of hyphae in a tissue section (with or without conidia) along with PCR positivity to Oo allows to state that the snake was affected by disease. On the other hand, ophidians with Oo negative molecular results, with or without macroscopic signs, are graded as “Not detected” instead of “Negative” because no histological absence of hyphae is provided. Although there is clear agreement among the classifications for Oo molecular positives with no gross signs and histological confirmation – such as NT4, categorised as “*Ophidiomyces* present” by Baker et al. (2019), “Detected” by Davy et al. (2021), and “Oo present” by Di Nicola et al. (2022a) –, our cases play different in these grading schemes. Substantial disagreement arises regarding the “confirmation” of the disease, which, according to Baker et al. (2019), requires the histological assessment of arthroconidia rather than solely hyphae, as in the other two systems. Moreover, (i) histological presence of hyphae with PCR Oo positivity and (ii) presence of gross signs with Oo molecular positivity, overlap within the same grade of “Apparent Ophidiomycosis” in the scheme proposed by Baker et al. (2019). The classification by Davy and colleagues (2021) is more inclusive, as it provides an outcome for each case without excluding negative results, which could be useful for prospective population studies where all cases need to be categorised. The classification by Di Nicola et al. (2022a) is more restrictive, as it excludes all cases with Oo negative molecular results. Its criteria were formulated to retrospectively review cases published in the literature and to avoid the inclusion of potential negative outcomes, resulting in a limited study cohort. Thus, this classification system seems to be better suited for describing and ranking the susceptibility or infection status at the taxon level rather than diagnosing individual cases. There is a need for scientists, veterinarians, and wildlife biologists to adopt a consistent approach for addressing cases of clinical ophidiomycosis and reach a clear consensus on grading schemes at a global level.

*Natrix tessellata* seems consistently susceptible to *Ophidiomyces* mycosis. A moult from Czech Republic was found positive for Oo (Franklinos et al. 2017), dice snakes with apparent ophidiomycosis (i.e. macroscopic lesions and molecular positivity) have been reported in Austria, Germany, Switzerland and Hungary (Blanvillain et al. 2022; Schüler et al. 2022), and a Swiss specimen labelled in 1961 was affected by lympho-histiocytic *Ophidiomyces* dermatitis (Origgi et al. 2022). *Natrix tessellata* has the highest prevalence of detection amongst the European species evaluated for Oo DNA by Blanvillain and colleagues (2022), driving hotspots in Switzerland. At the same time, both Clade I (European) and II (American) were detected from this species: Blanvillain et al. (2022) found ITS genotypes matching both Clade I and II as reported by Ladner and colleagues (2022); Origgi et al. 2022 reported a case in which Oo ITS, ACT (actin) and TEF (transcription elongation factor) genotypes were consistent with Clade II. Our findings support that the dice snake is particularly susceptible to clinical affection, confirming that it sheds the pathogen and may be highly infectious.

Although our sample pool is very limited, 3 out of 4 positive snakes from the Garda area were juveniles. Ophidiomycosis in neonate and young individuals seems to be a emerging hazard (Britton et al. 2019; Stengle et al. 2019). Nevertheless, in the US, this age class presented subtle or absent clinical signs (Britton et al. 2019; Harding et al. 2022), unlike the individuals affected by the disease on Lake Garda shores. Further studies should be directed at understanding whether Oo is able to provoke “silent death” in European snakes of this demographically important age class, and, indirectly, local extinction impacting demographic and population fitness (Di Nicola et al. 2022a).

Additional work can also include culturing of the collected material to obtain fungal isolates which can then be used for a complete phylogenetic analysis (e.g. Franklinos et al. 2017; Sun et al. 2021; Ladner et al. 2022). Although this was beyond the scope of the current study, further in-depth surveys in Italy should be performed and these would support phylogenetic analyses of the pathogen.

In conclusion, this study confirms for the first time the presence of Oo and its associated infection in live ophidians from northern Italy. The disease affects *N. tessellata* and is still present in the Italian territories after over a half a century, as confirmed in two *Natrix helvetica* museum specimens preserved in Switzerland from unknown Italian locations (Origgi et al. 2022). Furthermore, a fast and less expensive method for Oo monitoring was shown to have potential utility for future surveillance efforts.

## Electronic supplementary material

Below is the link to the electronic supplementary material.

Supplementary Material 1

## Data Availability

Data generated or analysed during this study are partially included in this published article and its supplementary information files. Representative sequences were deposited in the NCBI Nucleotide database under accession numbers OQ612704, OQ613488 and OQ607750. Raw data generated during and/or analysed during the current study are available from the corresponding author on reasonable request.
