# Supplementary material for: Pilot survey reveals ophidiomycosis in dice snakes Natrix tessellata from Lake Garda, Italy
Source: Vet Res Commun. 2023 Apr 29;47(3):1707–19. doi: 10.1007/s11259-023-10129-7 (PMC10485108; doi:10.1007/s11259-023-10129-7)
Supplement: Supplementary file 1 — Supplementary Material 1 [file 11259_2023_10129_MOESM1_ESM.docx]

**Supplementary Files**

**Pilot survey reveals ophidiomycosis in dice snakes *Natrix tessellata* from Lake Garda, Italy**

Daniele Marini^1,2,^*^,§^, Matteo R. Di Nicola^3,4,§^, Veronica Crocchianti^5^, Tommaso Notomista^6^, Daniel Iversen^7^, Luca Coppari^8^, Michela Di Criscio^1^, Vanessa Brouard^1^, Jean-Lou C.M. Dorne^9^, Joëlle Rüegg^1^, Maria Luisa Marenzoni^2^

**Affiliations**:

^1^ Department of Organismal Biology, Evolutionary Biology Centre, Uppsala University, Norbyvägen 18A, 75236 Uppsala, Sweden.

^2^ Department of Veterinary Medicine, University of Perugia, Via San Costanzo 4, 06126 Perugia, Italy.

^3^ IRCCS San Raffaele Hospital, Unit of Dermatology, Via Olgettina 60, 20132 Milan, Italy.

^4^ Asociación Herpetológica Española, Apartado de correos 191, 28911 Leganés, Madrid, Spain.

^5^ Service d’Anatomie Pathologique, VetAgro Sup, Campus Vétérinaire, 1 Avenue Bourgelat, 69280 Marcy l'Etoile, France.

^6^ Via Varano 1, 80053 Castellammare di Stabia, Italy.

^7^ Viale Giovanni Prati, 38066, Riva del Garda, Italy

^8^ Studio Naturalistico Hyla s.r.l., Via Baroncino, 11 - 06069 Tuoro sul Trasimeno (PG), Italy.

^9^ Methodology and Scientific Support Unit, European Food Safety Authority (EFSA), Via Carlo Magno 1A, 43126 Parma, Italy

*Corresponding author. Email: daniele.marini@ebc.uu.se

§These authors share first authorship

**Supplementary Fig. S1:** Adult *Natrix tessellata* photographed on 13th June 2015 on Garda lake showing signs consistent of ophidiomycosis. Photocredit: Daniel Iversen.


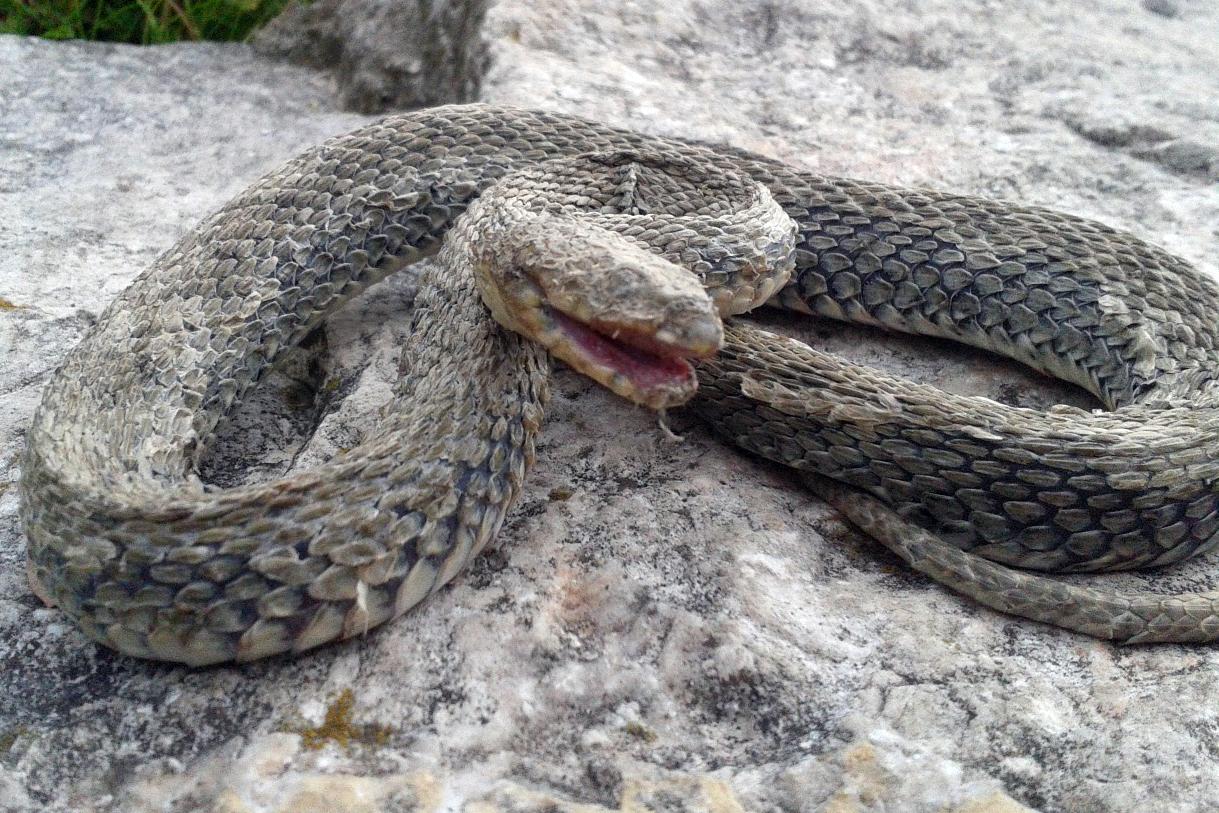


**Supplementary Fig. S2:** Bio-Rad CFX Maestro software showing ITS2 and nad1 melting curves and peaks from the same plate and the same positive control (POS0), amplified with the same cycling conditions.


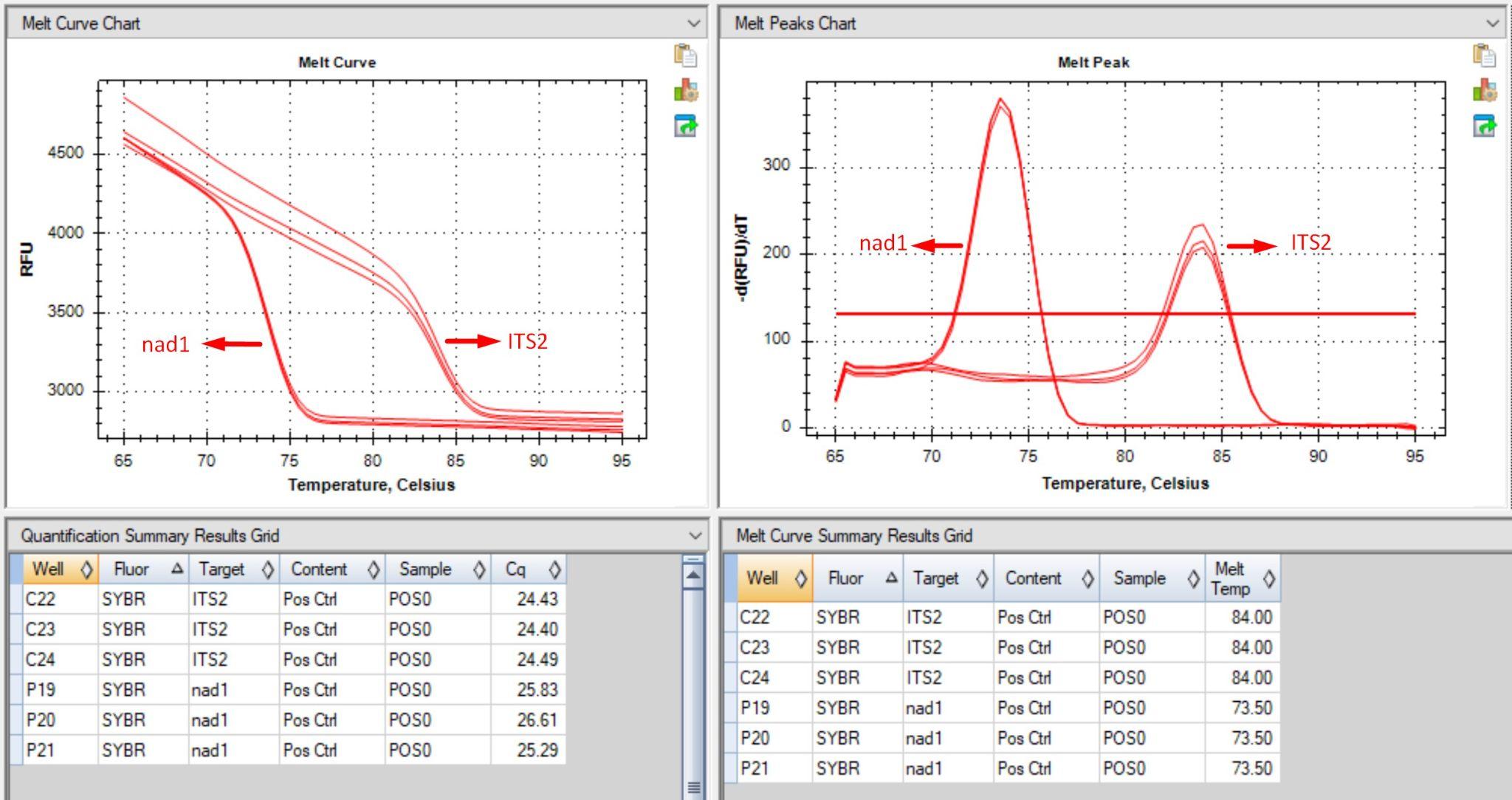


**Supplementary Fig. S3:** Efficiency standard curve constructed with the ITS2 primers in the real-time PCR with the SYBR Green detection system. The r2 value was 0.995 and the amplification efficiency was 102.7% [EFF =10^(-1/a)-1 resulted in 1.027 without outliers]. The assay linearity was conducted using a serial dilution of the positive control (DNA extracted from a tissue of a positive snake provided by F.C. Origgi). The serial four-fold dilution (DF = 1/4) with a starting positive control DNA concentration of 16 ng/µl yielded the concentrations (ng/µl) of 16, 4, 1, 0.25, 0.0625 and 0.0156 with average Ct values(±SD) of 21.97(±0.22), 23.63(±0.11), 26.00(±0.13), 27.55(±0.18), 29.36(±0.13) and 31.96(±0.12), respectively.


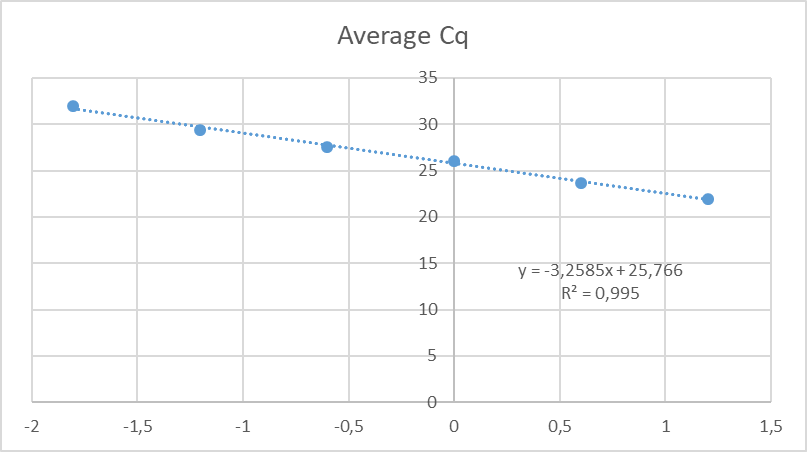


**Supplementary Figure S4:** Snapshot of scanned slide showing chronic epidermal inflammation in NT1 Oo infection. Superficial epidermal layers display severe necrosis and intralesional abundant hyphae effacing the normal epidermal structure. The basal layer is hyperplastic, dysplastic and minimally to mildly infiltrated by pigmented macrophages and degenerated heterophils. PAS stain.


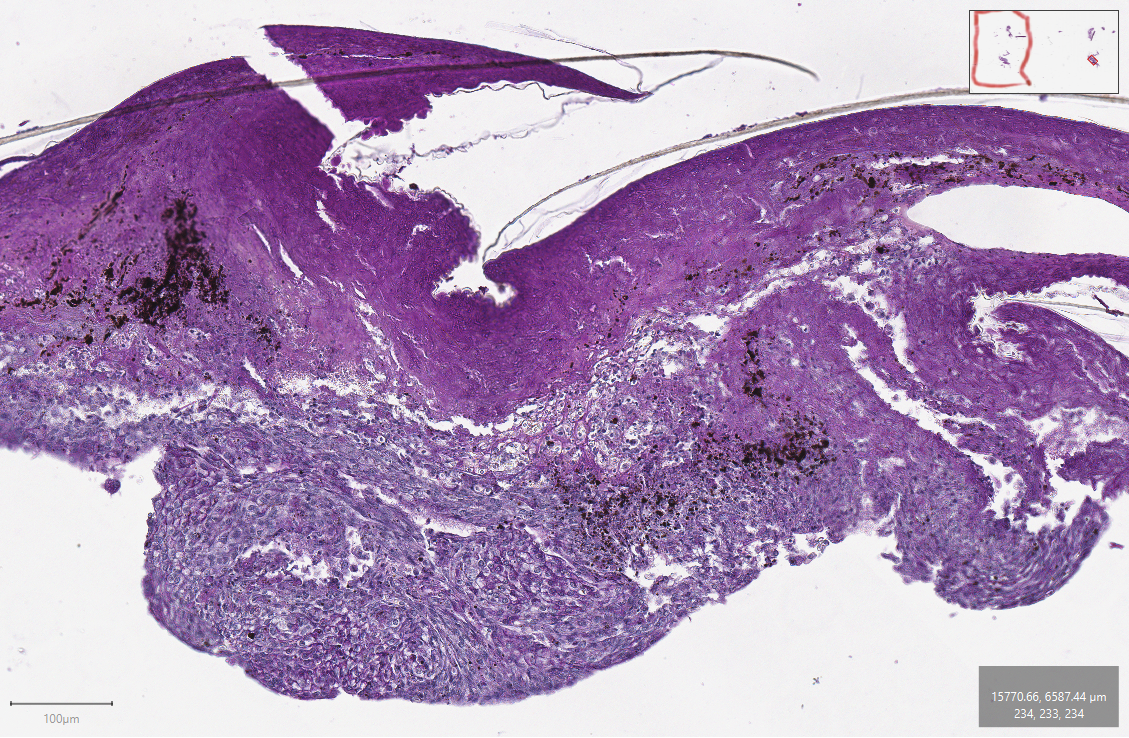


**Supplementary Table S1:** Concentrations (ng/µl) of DNA extracted from each sample measured with Nanodrop 2000c spectrophotometer (ThermoFisher, Carlsbad, CA, USA). Extracted DNA in bold yielded ITS2 positive results, and underlined ones represent those positive for nad1 (see Table S4). S(n): swab and, in parentheses, the identification number randomly assigned to one of the three swabs analyzed for each individual snake. T(n): tissue and identification number for the tissue sample. #: scale clip. H: used for histology. /: not available.

| **ID code** | **Finding condition** | **Sample type** | **DNA concentration (ng/µl) per sample** | | | | |
| --- | --- | --- | --- | --- | --- | --- | --- |
|  |  |  | S(1) | S(2) | S(3) | T(1) | T(2) |
|  | | | | | | | |
| CG21 | Dead (road-killed) | Tissue in 96% ethanol | / | / | / | 580.7 | / |
| CG01 | Dead (road-killed) | Frozen tissue | / | / | / | 973.3 | / |
| CG02 | Live | (3) Dry swabs | 21.3 | 7.7 | 18.9 | / | / |
| HV03 | Dead (road-killed) | Frozen tissue | / | / | / | 1686.2 | / |
| HVGU | Dead (road-killed) | Tissue in 96% ethanol | / | / | / | 1007.5 | / |
| HVPG | Live | (3) Dry swabs | 8.9 | 4 | 7.8 | / | / |
| BSS | Live | (3) Dry swabs | 12.9 | 8.1 | 17.4 | / | / |
| HV02 | Dead (road-killed) | Frozen tissue | / | / | / | 1057 | / |
| HV01 | Dead (road-killed) | Tissue in 96% ethanol | / | / | / | 830.4 | 486.4 |
| NHCE | Live | (3) Dry swabs | 17.9 | 19.6 | 5.9 | / | / |
| NH02 | Dead (road-killed) | Tissue in 96% ethanol | / | / | / | 232.1 | / |
| NH01 | Live | (3) Dry swabs | 8.9 | 17.55 | 6.9 |  |  |
| NH03 | Dead (road-killed) | Frozen tissue | / | / | / | 1597.9 | / |
| NT1 | Live | (3) Dry swabs + scale clip in 96% ethanol | 18.5 | **19.5** | **8.4** | # H | / |
| NT2 | Live | (3) Dry swabs + scale clip in 96% ethanol | **13.3** | **17.4** | 7 | # H | / |
| NT3 | Live | (3) Dry swabs + scale clip in 96% ethanol | 75.1 | **22.1** | **17.4** | # H | / |
| NT4 | Live | (3) Dry swabs + scale clip in 96% ethanol | 11.3 | 30.3 | 28.8 | # **113.7** | / |

**Supplementary Table S2:** PCR types and primers used in this study.

| **PCR type** | **Region** | **Primers** | **Primers reference** |
| --- | --- | --- | --- |
| Real-time PCR  (SYBR Green-based) | Internal transcribed spacer 2 (ITS2 within the rRNA gene complex - genomic) | Oo-rt-ITS-F:  5′ - GAGTGTATGGGAATCTGTTTC - 3′  Oo-rt-ITS-R:  5′ - GGTCAAACCGGAAAGAATG - 3′ | Bohuski E, Lorch JM, Griffin KM, Blehert DS (2015). TaqMan real-time polymerase chain reaction for detection of *Ophidiomyces ophiodiicola*, the fungus associated with snake fungal disease. BMC Vet Res 11(1), 1-10. https://doi.org/10.1186/s12917-015-0407-8 |
| Real-time PCR  (SYBR Green-based) | NADH dehydrogenase subunit 1 (nad1 - mitochondrial) | Oo-nad1-F:  5′ - ACTTGATTGTTTCTCTAGTC - 3′  Oo-nad1-R:  5′ - AGGGAAAGAAGCTCTAAC - 3′ | Lorch, JM, Price SJ, Lankton JS, Drayer AN (2021) Appendix 2. Confirmed cases of Ophidiomycosis in Museum specimens from as early as 1945, United States. Emerg Infect Dis 27. https://doi.org/10.3201/eid2707.204864 |
| Conventional PCR | D1-D2 region of the large subunit of the rRNA gene (genomic - broad-range panfungal PCR) | LSU266F:  5' - GAGTCGAGTTGTTTGGGAATGC - 3'  LSU654R:  5' - GGTCCGTGTTTCAAGACGG - 3' | Franklinos LH, Lorch JM, Bohuski E, Fernandez JRR, Wright ON, Fitzpatrick L, Petrovan S, Durrant C, Linton C, Baláž V, Cunningham AA, Lawson B (2017) Emerging fungal pathogen *Ophidiomyces ophiodiicola* in wild European snakes. Sci Rep-Uk 7:1-7. https://doi.org/10.1038/s41598-017-03352-1 |

**Supplementary Table S3**: Snake samples included in the study with detailed information. +: Positive. -: Negative. N.P.: not performed. /: Excluded.

| **ID code** | **Species** | **Free ranging/**  **Captive** | **Date** | **Locality** | **Coordinates** | **Age class** | **Finding condition** | **Gross signs** | **Sample type** | **Real-time PCR**  **(ITS2 - genomic)** | **Real-time PCR**  **(nad1 - mitochondrial)** | **PanFungal PCR**  **(D1-D2 - genomic)** | **Histology** | **Classification by Baker et al. (2019)** | **Classification by Davy et al. (2021)** | **Classification by Di Nicola et al. (2022a)** |
| --- | --- | --- | --- | --- | --- | --- | --- | --- | --- | --- | --- | --- | --- | --- | --- | --- |
|  | | | | | | | | | | | | | |  |  |  |
| CG21 | *Coronella girondica* | Free ranging | 2021-10-05 | Guzzano (Emilia-Romagna) | 44.37, 11.32 | Juvenile | Dead (road-killed) | Y | Tissue in 96% ethanol | - | N.P. | N.P. | N.P. | Possible Ophidiomycosis | Not detected | / |
| CG01 | *Coronella girondica* | Free ranging | 2021-11-11 | Genova (Liguria) | 44.40, 8.96 | Sub-adult | Dead (road-killed) | N | Frozen tissue | - | N.P. | N.P. | N.P. | / | Not detected | / |
| CG02 | *Coronella girondica* | Free ranging | 2021-02-02 | Bagno di Romagna (Emilia-Romagna) | 43.88, 11.89 | Adult | Live | N | (3) Dry swabs | - | N.P. | N.P. | N.P. | / | Not detected | / |
| HV03 | *Hierophis viridiflavus* | Free ranging | 2021-05-10 | Giussago (Lombardy) | 45.28, 9.15 | Sub-adult | Dead (road-killed) | N | Frozen tissue | - | N.P. | N.P. | N.P. | / | Not detected | / |
| HVGU | *Hierophis viridiflavus* | Free ranging | 2021-09-29 | Guidonia Montecelio (Latium) | 41.97, 12.71 | Adult | Dead (road-killed) | Y | Tissue in 96% ethanol | - | N.P. | N.P. | N.P. | Possible Ophidiomycosis | Not detected | / |
| HVPG | *Hierophis viridiflavus* | Free ranging | 2021-05-13 | Marsciano (Umbria) | 42.97, 12.35 | Juvenile | Live | Y | (3) Dry swabs | - | N.P. | N.P. | N.P. | Possible Ophidiomycosis | Not detected | / |
| BSS | *Hierophis viridiflavus* | Free ranging | 2021-05-19 | Sorico (Lombardy) | 46.21, 9.43 | Adult | Live | Y | (3) Dry swabs | - | N.P. | N.P. | N.P. | Possible Ophidiomycosis | Not detected | / |
| HV02 | *Hierophis viridiflavus* | Free ranging | 2021-02-09 | Moglia (Lombardy) | 44.93, 10.93 | Adult | Dead (road-killed) | N | Frozen tissue | - | N.P. | N.P. | N.P. | / | Not detected | / |
| HV01 | *Hierophis viridiflavus* | Free ranging | 2021-03-10 | Villanterio (Lombardy) | 45.22, 9.36 | Adult | Dead (road-killed) | N | Tissue in 96% ethanol | - | N.P. | N.P. | N.P. | / | Not detected | / |
| NHCE | *Natrix helvetica cetti* | Free ranging | 2021-04-03 | Sinnai (Sardinia) | 39.29, 9.43 | Adult | Live | N | (3) Dry swabs | - | N.P. | N.P. | N.P. | / | Not detected | / |
| NH02 | *Natrix helvetica sicula* | Free ranging | 2021-09-01 | Montevecchia (Lombardy) | 45.71, 9.37 | Sub-adult | Dead (road-killed) | N | Tissue in 96% ethanol | - | N.P. | N.P. | N.P. | / | Not detected | / |
| NH01 | *Natrix helvetica sicula* | Free ranging | 2021-04-25 | Caselette (Piedmont) | 45.11, 7.47 | Sub-adult | Live | N | (3) Dry swabs | - | N.P. | N.P. | N.P. | / | Not detected | / |
| NH03 | *Natrix helvetica sicula* | Free ranging | 2021-07-01 | Giussago (Lombardy) | 45.29, 9.15 | Adult | Dead (road-killed) | N | Frozen tissue | - | N.P. | N.P. | N.P. | / | Not detected | / |
| NT1 | *Natrix tessellata* | Free ranging | 2021-03-02 | Riva del Garda (Province of Trento) | 45.88, 10.85 | Juvenile | Live | Y | (3) Dry swabs + scale clip | + | + | Oo not amplified | +  (hyphae and conidia) | Confirmed Ophidiomycosis | Ophidiomycosis | Ophidiomycosis and Oo shedder |
| NT2 | *Natrix tessellata* | Free ranging | 2021-03-02 | Riva del Garda (Province of Trento) | 45.88, 10.85 | Juvenile | Live | Y | (3) Dry swabs + scale clip | + | + | + | +  (hyphae and conidia) | Confirmed Ophidiomycosis | Ophidiomycosis | Ophidiomycosis and Oo shedder |
| NT3 | *Natrix tessellata* | Free ranging | 2021-03-02 | Riva del Garda (Province of Trento) | 45.88, 10.85 | Juvenile | Live | Y | (3) Dry swabs + scale clip | + | + | + | +  (hyphae) | Apparent Ophidiomycosis | Ophidiomycosis | Ophidiomycosis |
| NT4 | *Natrix tessellata* | Free ranging | 2021-03-02 | Riva del Garda (Province of Trento) | 45.88, 10.85 | Adult | Live | N | (3) Dry swabs + scale clip | + | + | + | N.P. | *Ophidiomyces* present | Detected | Oo present |

**Supplementary Table S4:** Triplicate average of threshold cycle (Ct), Melting temperature and End RFU (relative fluorescence unit) values across Real-time PCR runs of Lake Garda samples. S(n): swab and, in parentheses, the identification number randomly assigned to one of the three swabs performed for each individual. T: tissue (in this case, a scale clip). (+): Positive. (-): Negative. *: 2 replicates considered (i.e. positives) out of three for average calculations.

| **Target** | **Sample type** | **Sample** | **Average Ct values (average pos ctrl)** | **Average Melting Temperature (average pos ctrl)** | **Average End RFU**  **(average pos ctrl)** | **Call (RFU End-Point)** |
| --- | --- | --- | --- | --- | --- | --- |
| ITS2 | S(1) | NT1 | N/A | N/A | N/A | (-) |
| ITS2 | S(1) | NT2 | 25.41 (23.19) | 83 (84) | 1070 (1772) | (+) |
| ITS2 | S(1) | NT3 | N/A | N/A | N/A | (-) |
| ITS2 | S(1) | NT4 | N/A | N/A | N/A | (-) |
| ITS2 | S(2) | NT1 | 29.05 (23.71) | 83 (84) | 1341 (2062) | (+)* |
| ITS2 | S(2) | NT2 | 25.43 (23.71) | 83 (84) | 1556 (2062) | (+) |
| ITS2 | S(2) | NT3 | 24.36 (23.71) | 83 (84) | 1960 (2062) | (+) |
| ITS2 | S(2) | NT4 | N/A | N/A | N/A | (-) |
| ITS2 | S(3) | NT1 | 27.15 (24.20) | 83.5 (84) | 1230 (1635) | (+) |
| ITS2 | S(3) | NT2 | N/A | N/A | N/A | (-) |
| ITS2 | S(3) | NT3 | 23.93 (24.20) | 83.5 (84) | 1457 (1635) | (+) |
| ITS2 | S(3) | NT4 | N/A | N/A | N/A | (-) |
| ITS2 | T | NT4 | 29.67 (25.63) | 83.5 (84) | 1589 (2365) | (+) |
| nad1 | S(2) | NT1 | 30.36 (29.63) | 73 (73.5) | 496 (1118) | (+)* |
| nad1 | S(1) | NT2 | 25.49 (29.63) | 73.5 (73.5) | 888 (1118) | (+)* |
| nad1 | S(3) | NT3 | 24.71 (29.63) | 73 (73.5) | 1080 (1118) | (+) |
| nad1 | T | NT4 | 31.66 (26.65) | 73.5 (73.5) | 913 (1973) | (+) |

**Supplementary Table S5:** Sequences obtained for each positive individual and each primer set. Sequences selected and deposited in the NCBI Nucleotide database show accession numbers under the column GenBank Accession Number. Primer sequences or their reverse complements are not excised from the whole sequences and are highlighted in bold. The sequence underlined in the ITS2 sequences corresponds to the probe marked with 6-carboxyfluorescein (FAM) and Black Hole Quencher^®^-1 (BHQ-1) used during TaqMan real-time PCR (5′-(FAM)TCTCGCTCGAAGACCCGATCG(BHQ-1)-3′ - Bohuski et al., 2015). /: not submitted.

| **Individual** | **Region** | **Obtained sequence (5’-3’; strand plus/plus)** | **Percent identity (BLAST) with first Oo sequence in Genbank (in square brackets)** | **GenBank**  **Accession Number** |
| --- | --- | --- | --- | --- |
| NT1 | ITS2 | **GAGTGTATGGGAATCTGTTTC**TGTCTCGCTCGAAGACCCGATCGGCGCC-GTCGTC-ACCCCC**CATTCTTTCCGGTTTGACC** | 97.56% (80/82; 2/82 gaps) [KF225599.1] | / |
|  | nad1 | **ACTTGATTGTTTCTCTAGTC**TTAATTTAGCTTTAAAAACTAGTTTTCTTATCTTTGTTTTTATTTGA**GTTAGAGCTTCTTTCCCT** | 100% (85/85) [MW358097.1] | OQ613488 |
|  | D1-D2 | TTCTTCT-AAGCTAAATATTGGCCAGAGACCGATAGCGCACAAGTAGAGTGATCGAAAGATGAAAAGCACTTTGGAAAGAGAGTTAAAAAGCACGTGAAATTGTTGAAAGGGAAGGGATTGCAACCAGACTTGCTCGCGGTGTTCCGCCGGTCTTCTGACCGGTCTACTCGCCGCGTTGCAGGCCAGCATCGTCTGGTGCCGCTGGATAAGACTTGAGGAATGTAGCTCCCTCGGGAGTGTTATAGCCTCTTGTCATGCAGCCAGCGCCGGGCGAGGTCCGCGCTCGGCTAGGATGC | Oo not amplified: *Cladosporium* sp. - 98,99% (294/297; 1/297 gap) [*Cladosporium sphaerospermum* GQ901955.1; *Cladosporium halotolerans* LC414352.1] | / |
| NT2 | ITS2 | **GAGTGTATGGGAATCTGTTTC**TGTCTCGCTCGAAGACCCGATCGGCGCC-GTCGTC-ACCCCC**CATTCTTTCCGGTTTGACC** | 97.56% (80/82; 2/82 gaps) [KF225599.1] | / |
|  | nad1 | **ACTTGATTGTTTCTCTAGTC**TTAATTTAGCTTTAAAAACTAGTTTTCTTATCTTTGTTTTTATTTGA**GTTAGAGCTTCTTTCCCT** | 100% (85/85) [MW358097.1] | / |
|  | D1-D2 | AATTTCATCTAAAGCTAAATATTGGCCGGAGACCGATAGCGCACAAGTAGAGTGATCGAAAGGTTAAAAGCACCTTGAAAAGGGAGTTAAATAGCACGTGAAATTGTTGAAAGGGAAGCGCTTGCAACCAGACTCGGTTCCGGGGGCTCAGCGGGCATGCGTGCCCGTGTACTCCCCCGTCTCCGGGCCAGCATCAGTTCGAGCGGCCGGTCAAAGGCCCCGGGAACGTATCATCCTCCGGGATGCCTTATAGCCCGGGGTGCAATGCGGCCAGCTCGGACTGAGGAACGCGCCTCGGCACGGATGCTGGCATAATGGTTGTAAGCGGC**CCGTCTTGAAACACGGACC** | 100% (348/348) [KF225601.1] | / |
| NT3 | ITS2 | **GAGTGTATGGGAATCTGTTTC**TGTCTCGCTCGAAGACCCGATCGGCGCC-GTCGTC-ACCCCC**CATTCTTTCCGGTTTGACC** | 97.56% (80/82; 2/82 gaps) [KF225599.1] | / |
|  | nad1 | **ACTTGATTGTTTCTCTAGTC**TTAATTTAGC-TTAAAAACTAGTTTTCTTATCTTTGTTTTTATTTGA**GTTAGAGCTTCTTTCCCT** | 98.82% (84/85; 1/85 gap) [MW358097.1] | / |
|  | D1-D2 | **GAGTCGAGTTGTTTGGGAATGC**AGCTCTAAGTGGGTGGTAAATTTCATCTAAAGCTAAATATTGGCCGGAGACCGATAGCGCACAAGTAGAGTGATCGAAAGGTTAAAAGCACCTTGAAAAGGGAGTTAAATAGCACGTGAAATTGTTGAAAGGGAAGCGCTTGCAACCAGACTCGGTTCCGGGGGCTCAGCGGGCATGCGTGCCCGTGTACTCCCCCGTCTCCGGGCCAGCATCAGTTCGAGCGGCCGGTCAAAGGCCCCGGGAACGTATCATCCTCCGGGATGCCTTATAGCCCGGGGTGCAATGCGGCCAGCTCGGACTGAGGAACGCGCCTCGGCACGGATGCTGGCATAATGGTTGTAAGCGGC**CCGTCTTGAAACACGGACC** | 100% (388/388) [KF225601.1] | OQ607750 |
| NT4 | ITS2 | **GAGTGTATGGGAATCTGTTTC**TGTCTCGCTCGAAGACCCGATCGGCGCCCGTCGTCAACCCCC**CATTCTTTCCGGTTTGACC** | 100% (82/82) [KF225599.1] | OQ612704 |
|  | nad1 | **ACTTGATTGTTTCTCTAGTC**TTAATTTAGCTTTAAAAACTAGTTTTCTTATCTTTGTTTTTATTTGA**GTTAGAGCTTCTTTCCCT** | 100% (85/85) [MW358097.1] | / |
|  | D1-D2 | CCAGCATCAGTTCGAGCGGCCGGTCAAAGGCCCCGGGAACGTATCATCCTCCGGGAGGCCTTATAGCCCGGGGTGCAATGCGGCCAGCTCCGGACCGAGGTAACGCGCCTCG | 96.43% (108/112; 2/112 gaps) [LT607735.1; KF225601.1] | / |
